# Designing Epitope Ensemble Vaccines against TB by Selection: Prioritizing Antigens using Predicted Immunogenicity

**DOI:** 10.6026/97320630013220

**Published:** 2017-07-31

**Authors:** Jaymisha Mistry, Darren R Flower

**Affiliations:** 1 School of Life and Health Sciences, Aston University, Aston Triangle, Birmingham, United Kingdom, B4 7ET.

**Keywords:** Designing epitope, Ensemble vaccines, TB, antigens, immunogenicity

## Abstract

Tuberculosis (TB) is a global health burden, and a major cause of mortality and morbidity in West Africa. Here, we select key conserved pathogen epitopes of proven immunogenicity to form a potential TB epitope ensemble vaccine. We compared two vaccine formulations: one comprising class I epitopes from the 13 most prevalent class I epitope-bearing antigens and class II epitopes deriving from the 20 most prevalent class II epitope-bearing antigens and another consisting of epitopes derived solely from 5 antigens identified as the most immunogenic by VaxiJen. In the prevalence analysis, 279 class I and 561 class II epitopes were collected and a subset selected using our published methodology, yielding 32 conserved epitopes. Combining 9 conserved epitopes gave a putative vaccine with predicted population coverage (PPC) over 95%. This consists of ISSGVFLLK, AVAGAAILV, WYYQSGLSI, YQSGLSIVM, MPVGGQSSF, QSSFYSDWY, WDINTPAFEWYYQSGLSIVM, DAPLITNPGGLLEQAVAVEE and NQAVTPAARALPLTSLTSAA. 5 immunogenic antigens VaxiJen-identified yielded 187 epitopes, which we again analyzed using published protocol. This identified 11 conserved epitopes. From this set the highest PPC value (<85%) was obtained by combining: GQQYQAMSAQAAAFH, DDIKATYDKGILTVSVAVSE and AVAGAAILV. We conclude that prioritizing epitope selection using predicted immunogenicity alone is likely to be unduly restrictive and is currently not an optimal or advisable strategy in the design of epitope ensemble vaccines.

## Background

Mycobacterium Tuberculosis (TB) is a global health burden with an estimated 2015 death toll of 1.8 million, with 10.5 million new TB cases [1]. TB most commonly presents as a pulmonary disease transmitted via droplet inhalation resulting in symptoms including persistent cough, fever, and night sweats [2]. Some individuals can clear the disease, others will be asymptomatic with latent TB, and a proportion will suffer active TB. The slow rate of TB growth, combined with its complex pathogenesis and ability to lay dormant, poses as a major challenge to developing effective treatments. Vaccine development currently remains a priority, especially in developing countries where it is endemic, such as Africa, India, and Indonesia [3].

The immune response against TB predominantly involves T cell mediated immunity, specifically CD4+ and CD8+ T cells, which bind to complexes of class I or class II MHC bound to specific epitopes. Once activated, both CD4+ and CD8+ cells secrete cytokines inducing an immune response. CD8+ T cells also mediate cytotoxicity and the lysis of infected cells.

The Bacillus Calmette-Guérin (BCG) anti-TB vaccine has been used for disease prophylaxis and control of TB progression for over 80 years. BCG is an attenuated form of mycobacterium bovis and is currently the only anti-TB vaccine in use worldwide. However, BCG has a well-attested variable efficacy (0-80%) in adult populations presenting with pulmonary TB [4], limiting its ability to prevent TB transmission. Thus the need for alternatives is clear.

Several live vaccines are currently in development against TB including recombinant BCG VPM1002 and MTBVAC [5]. A key issue with live vaccines is their capacity to revert to a pathogenic form. This is a danger for immunocompromised individuals. Viral vector-based vaccines include that based on the Ankara virus (MVA), modified to express TB antigen 85A, and an adenovirus expressing the mycobacterial antigens 85A, 85B and TB10.4, known as Crucell-Ad35/AERAS-402 vaccine [6]. Problems include the reduced efficacy of any resulting immunity due to previous exposure to the vector. There are several subunit vaccines being developed including the H1 vaccine, which combines 85B and ESAT-6 antigens, H4 vaccine which combines antigens 85B and TB10.4, and M72 which combines antigens 39A and 32A. The inability of subunit vaccines to induce long-term immunity necessitates multiple administrations or the inclusion of adjuvants.

Here, we seek to identify pathogen epitopes able of form an epitope ensemble vaccine against TB. The complete genome sequence of Mycobacterium Tuberculosis H37Rv strain, comprising over 4 million base pairs and approximately 4,000 genes [7], has helped in the search of vaccines against TB. An ideal vaccine would include highly conserved immunogenic epitopes with wide population coverage. Our recent studies exemplify this approach against viruses: Hepatitis C [8] and influenza [9]. Here we extend such work by addressing Mycobacterium Tuberculosis, a much larger and more complex bacterial pathogen. Specifically, we compared a vaccine comprised of epitopes from the most prevalent epitope-bearing antigens with one comprising epitopes deriving solely from antigens identified as most immunogenic by VaxiJen [10,11].

## Methodology

### Vaccine Design

**Figure 1:**
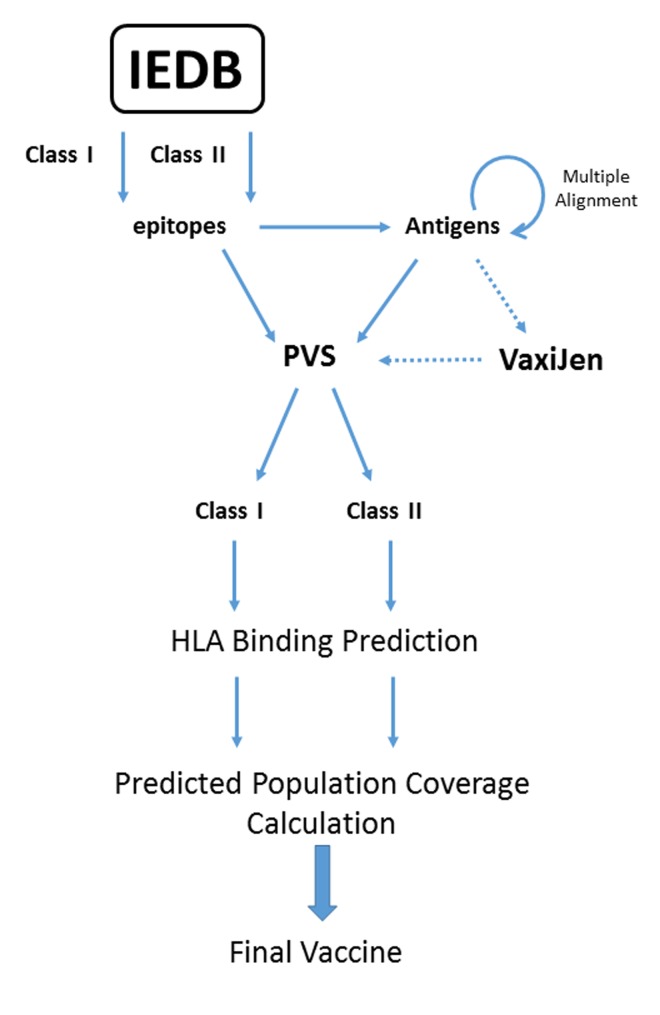
Protocol of epitope selection.

We applied an epitope ensemble vaccine design protocol already described [8,9] to TB. Epitopes were extracted from the Immune Epitope Database and analysis resource (IEDB; URL: http://www.iedb.org/). Search criteria: positive T cell assays, human host only. Full protein sequences of epitope-bearing antigens were extracted from NCBI (URL: https://www.ncbi.nlm.nih.gov/protein/) and searched using BLAST-P (URL: https://blast.ncbi.nlm.nih.gov/Blast.cgi) against the Protein Reference Sequences (Refseq_protein). To reduce bias, 10 sequences with high coverage and low identity were used to generate multiple sequence alignments. Sequence variability of the resulting alignments was calculated using the Protein Variability Server (PVS; URL: http://imed.med.ucm.es/PVS/), with a minimum fragment length of 9 and a variability threshold of 0.5. Class I and Class II binding profiles were calculated using IEDB׳s consensus method for class I (URL: http://tools.immuneepitope.org/mhci/) and class II (URL: http://tools.immuneepitope.org/mhcii/). Only epitopes with a predicted IC50 < 500nM were retained. Predicted population coverage (PPC) was calculated using the IEDB PPC tool (http://tools.iedb.org/tools/population/iedb_input) for two populations of interest: West Africa and the World. See Figure 1.

### Immunogenicity Prediction

TB Antigens were processed using VzxiJen (URL: http://www.ddgpharmfac.net/vaxijen/Vaxijen.html), which predicts immunogenicity scores, allowing classification of antigens as either an antigen capable of inducing protective immunity or as a non-antigen [10,11]. Bacteria were selected as the target organism, with the threshold set at 0.5. The five top scoring antigens for class I and class II were then processed as above. See Figure 1.

## Discussion

Two sets of epitope-bearing antigens were collected. The first dataset comprised proteins with greater than 10 epitopes. There were 13 Class I epitope-bearing antigens (Ag85B (59 class I epitopes), Putative ATP-dependent 6-phosphofructokinase isozyme 2 (28 epitopes), Alanine and proline-rich secreted protein Apa (26), Probable membrane protein Rv1733c (26), ESAT-6-like protein EsxB (23), ESAT-6-like protein EsxH (19), Low molecular weight T-cell antigen (17), 6 kDa early secretory antigenic target (16), Lipoprotein LpqH (14), PPE68 (14), Ag85A (13), Alpha-crystallin (13), MPT64 (11)) and 20 class II-bearing antigens (Ag85B (55 class II epitopes), 6 kDa early secretory antigenic target (55 epitopes), ESAT-6-like protein EsxB (45), 60 kDa chaperonin 2 (42), Uncharacterized PPE family protein PPE19 (36), Ag85A (34), Putative ATP-dependent 6- phosphofructokinase isozyme 2 (32), Alanine and proline-rich secreted protein Apa (30), Phosphate-binding protein PstS 1 (29), Uncharacterized PPE family immunogenic protein PPE68 (27), MPT64 (24), Alpha-crystallin (22), Lipoprotein LpqH (21), Probable membrane protein Rv1733c (21), MPT70 ( 19), 10 kDa chaperonin (18), MPT83 (15), Putative phthiocerol dimycocerosate transporter LppX (12), ESAT-6-like protein EsxH (12), MPT63 (12).

PVS identified conserved epitopes. From the 13 class I epitopebearing antigens, only 9 had conserved epitopes. Overall, 22 conserved epitopes were found. The greatest number of epitopes was collected from diacylglycerol acyltransferase Ag85B (11), followed by lipoprotein LpqH (3), diacylglycerol acyltransferase Ag85A (2), alanine and proline-rich secreted protein Apa (2), PPE68 (1), ATP-PFK 2 (1) and alpha-crystallin (1). From the 20 class I epitope-bearing antigens, only 5 had a total of 11 conserved epitopes: ATP-PFK 2 (1), alanine and proline-rich secreted protein Apa (1), lipoprotein LpqH (1), diacylglycerol acyltransferase Ag85B (3), PPE19 (5).

The second dataset comprised antigens with 3 or more epitopes for analysis using VAXIJEN. For class I, 22 antigens were identified. Antigens with the top 5 immunogenicity scores were identified: Lipoprotein LpqH (14 epitopes; score: 1.1666), Isoniazid-induced protein (3; 0.8784), ESAT-6-like protein EsxB (23, 0.8095), Alpha-crystallin (13; 0.8067), PPE68 (14; 0.6434). For class II, 61 antigens were identified. Antigens with the top 5 immunogenicity scores were identified: PE_PGRS14 (3 epitopes; score: 1.9588), Lipoprotein LpqH (21; 1.1666), ESAT-6-like protein EsxB (45; 0.8095), Alpha-crystallin (22; 0.8067); PstS 1 (29; 0.7270). PVS identified conserved epitopes: for class I only 4 antigens had conserved epitopes, returning a total of 6 epitopes, and for class II only 3 of the 5 antigens had conserved epitopes, returning a total of 5 epitopes.

For both VaxiJen and non-VaxiJen analysis, combinations of epitopes were assessed for population coverage. For the high prevalence set, non-VaxiJen analysis, the highest PPC value found was 98.75 (global) and 95.60 (West Africa). For the VaxiJen analysis, the highest PPC value found was 83.79 (global) and 68.62 (West Africa). See Table 1.

Compared to the unrestricted analysis, results from VaxiJen ranking were disappointing. Due to the small number of conserved epitopes identified, limited combinations could be undertaken. The conserved epitopes from VAXIJEN have similar HLA binding profiles. For class I, A*02:06, A*02:01 and A*02:03 were the most common restrictions seen. The limited variation in binding profiles contributed to the low PPC values seen. The highest combined PPC values for both subsets contained the class I conserved epitope AVAGAAILV, derived from lipoprotein LpqH, consistent with the epitope being highly immunogenic and promiscuous.

## Conclusion

In this vaccine design exercise, we sought to limit selection to epitopes from major highly immunogenic antigens. Such constrained selection should concentrate the immune response on antigens more likely repeatedly to evoke strong responses in vivo. As an alternative to using all experimentally verified epitopes available, we used VaxiJen - the first and, ostensibly, the only on-line server for whole protein immunogenicity prediction - to rank antigens in terms of potential efficacy. However, prioritizing antigens using VaxiJen did not prove effective. This identifies a shortcoming of this method, due in no small part to the dearth of adequate training data. This is something we will seek to rectify in due course. Prevalence analysis, however, allowed for the total pool of available epitopes to be tractable yet large enough to generate an optimal hypothetical vaccine candidate. Epitope combinations derived through epitopeprevalence analysis had sufficiently high population coverage to generate effective potential universal epitope ensemble vaccines. However, due to the low immunogenicity traditionally associated with peptide vaccines, such candidates are likely to require the addition of adjuvants. Overall, we have shown how computational approaches can identify a potential ensemble vaccine against a major bacterial pathogen by selecting epitopes of known, validated immunogenicity with broad population coverage. These will now need experimental validation to determine the efficacy and safety of the vaccine in live subjects.

## Figures and Tables

**Table 1:** Comparison of Vaccines in terms of Predicted Population Coverage

EPITOPE COMBINATION	OVERAL HLA BINDING PROFILE	Predicted Population Coverage WORLD (%)	Predicted Population Coverage WEST AFRICA (%)
Prevalence Analysis	A*30:01, A*31:01, A*11:01, A*03:01, A*68:01, A*02:06, A*68:02, A*02:03, A*02:01, A*23:01, A*24:02, B*15:01, B*35:01, B*40:01, B*53:01, B*07:02, A*01:01, A*30:02, DRB1*01:01, DQA1*01:02/DBQ1*06:02, DRB1*13:02, DQA1*05:01/DQB1*03:01, DQA1*04:01/DQB1*04:02, DQA1*03:01/DQB1*03:02, DRB4*01:01, DRB1*09:01, DRB1*04:01, DRB1*08:01, DRB4*01:01, DPA1*01/DPB1*04:01, DPA1*01:03/DPB1*02:01, DPA1*02:01/DPB1*01:01, DPA1*03:01/DPB1*04:02, DRB1*04:05, DRB1*07:01, DRB1*08:02, DRB1*11:01, DRB1*15:01, DRB5*01:01	98.79	95.6
ISSGVFLLK, AVAGAAILV, WYYQSGLSI, YQSGLSIVM, MPVGGQSSF, QSSFYSDWY, DAPLITNPGGLLEQAVAVEENQAVTPAARALPLTSLTSAA, GLQHISSGVFLLKASVREL			
VaxiJen Analysis	DQA1*01:02/DQB1*06:02, DQA1*04:01/DQB1*04:02, DQA1*05:01/DQB1*03:01, DRB1*01:01, DRB1*04:01, DRB1*04:05, DRB1*09:01, DRB1*11:01, DRB1*15:01, DRB4*01:01, DRB5*01:01, DRB1*01:01, DRB1*04:05, DRB1*07:01, DRB1*08:02, DRB1*13:02, DRB1*09:01, A*02:06, A*68:02, A*02:03, A*02:01	83.29	68. 62
GQQYQAMSAQAAAFH, DDIKATYDKGILTVSVAVSE, AVAGAAILV
